# Rising pharmaceutical innovation in the Global South: a landscape study

**DOI:** 10.1186/s40545-023-00669-3

**Published:** 2023-11-27

**Authors:** Marcela Vieira, Tatiana Andia, Obaida Karim, Sanjida Ahmed Srishti, Sebastian Alfonso Pineda, Adrian Alonso Ruiz, Kaitlin Large, Yiqi Liu, Suerie Moon, Nahitun Naher, Azizah Siddiqui, Syed Masud Ahmed

**Affiliations:** 1 Global Health Centre, Geneva Graduate Institute, Maison de La Paix, Chemin Eugène-Rigot 2, 1202 Geneva, Switzerland; 2 Universidad de los Andes, Cra. 1 #18a-12, La Candelaria, Bogotá, Cundinamarca Colombia; 3 BRAC James P Grant School of Public Health, BRAC University, 7/8/10th Floor, Medona Tower, 28 Mohakhali Commercial Area, Bir Uttom A K Khandakar Road, Dhaka, 1213 Bangladesh

**Keywords:** Pharmaceutical innovation, Global South, Research and development, Access to medicines

## Abstract

**Background:**

There is growing interest in pharmaceutical innovation in low- and middle-income countries (LMICs), but information on existing activities, capacities, and outcomes is scarce. We mapped available data at the global level, and studied the national pharmaceutical innovation systems of Bangladesh and Colombia to shed light on pharmaceutical research and development (R&D) in the Global South, including challenges and prospects, to help fill existing knowledge gaps.

**Methods:**

We gathered and analyzed data from three types of sources: literature, semi-structured interviews with key informants, and publicly available data on R&D funding, R&D scientific capacity measured by human resources, and clinical trial activities.

**Results:**

Pharmaceutical R&D activities are occurring in many LMICs, but 16 countries have emerged as frontrunners. Investment in R&D in LMICs has increased in the past decade, particularly from middle-income countries (MICs). Capacity is also growing, with an increase in the number of research organizations and the amount of funding available from external sources. The total number of clinical trials and the proportion of trials in LMICs increased markedly, and there is also growing activity in the earlier, more innovative and riskier Phase 1 and 2 trials. Non-commercial entities comprise the majority of clinical trial funders and sponsors in LMICs. Finally, investments have borne fruit, as indicated by a number of innovative medicines developed in LMICs. The Bangladesh and Colombia country studies showed that there is still a need for both targeted R&D policies to strengthen capacities in the pharmaceutical sector, and more government support to overcome the challenges of a lack of funding and coordination among different actors.

**Conclusions:**

By triangulating between the data sources, it was possible to paint a broad picture of who was involved in pharmaceutical R&D in LMICs, in which particular countries, for which diseases, in which R&D phases, and with what results—as well as how these trends have changed over time. Prioritizing pharmaceutical R&D is an important strategy for better meeting health needs. The trendlines are promising, but focused attention is still needed to realize the potential for greater innovation in the Global South.

## Background^1^

Over the past several decades, the pharmaceutical research and development (R&D) system has evolved to include greater participation from countries beyond those traditionally considered innovation hubs, such as the United States and a few Western European countries [39]. However, information about pharmaceutical innovation in the Global South more broadly, is still scarce. A better understanding of the roles, capacities, and outcomes of non-traditional actors might help to address some of the challenges that the system has built by design, such as unaffordable prices, unmet health needs, and globally unequal access to the outcomes of pharmaceutical innovation [25].

The COVID-19 pandemic has shed new light on long-standing problems, with growing calls to end both disparities in access to medicines and the related concentration of knowledge and technology in a few countries [38]. In the wake of the pandemic, there is increased interest in strengthening global R&D capacity, especially in the Global South. However, the lack of sufficient information about global R&D and manufacturing capacities limits countries’ability to introduce policies and actions to address innovation and access needs. Given the complexity of pharmaceutical R&D, it is a major challenge to maintain comprehensive, updated information about the different actors and activities dispersed throughout the global ecosystem [2].

To fill this information gap, we initiated a research collaboration between the Global Health Centre (GHC), Geneva Graduate Institute in Switzerland, the James P Grant School of Public Health, BRAC University in Bangladesh, and the Universidad de los Andes in Colombia, to further our collective understanding of pharmaceutical R&D activities in the Global South. Three research reports were produced as part of this collaboration: (i) one focused on pharmaceutical R&D in Bangladesh, led by BRAC University [17], (ii) one focused on pharmaceutical R&D in Colombia, led by Universidad de los Andes [2], and (iii) one focused on pharmaceutical R&D in low- and middle-income countries (LMICs) generally, led by the Geneva Graduate Institute [39]. Mapping, gathering, and analyzing data from Bangladesh and Colombia adds in-depth information to the general mapping of LMICs, enabling a cross-regional perspective of South Asia and Latin America. In addition, it provides additional information and perspective based on the experiences of two countries that are less discussed in the literature, given that studies about pharmaceutical innovation in the Global South most often analyze larger, more established countries in the field, such as China, India, and Brazil (see literature review section below). In the present article, we summarize the main findings from these three reports and analyze the data from a comparative perspective. The full reports are available at the Knowledge Portal for Innovation and Access to Medicines (www.knowledgeportalia.org).

## Methods

We used a mixed method research design for synthesizing evidence from qualitative and quantitative data [8]. The three research teams aimed to examine the available evidence on pharmaceutical R&D capacities and activities in LMICs at two different levels: whereas BRAC and Andes focused on their respective national levels, the team at the GHC focused their activities across the LMIC income group. The teams applied three distinct methods to collect and analyse data: a literature review, semi-structured interviews with experts and a descriptive analysis of publicly available information on R&D funding, R&D capacities, and clinical trial activities. For the literature review, the research teams conducted searches using keywords, such as “pharmaceutical”, "health", “innovation”, “research and development”, “product development”, “global south”, “developing countr*”, “low middle-income countr*”, "Bangladesh", and "Colombia" in major databases, including PubMed, SciELO, Global Index Medicus, Google Scholar, Scopus, Research4life, ScienceDirect, and Wiley, from the earliest available literature until mid-2022. The search was confined to articles written in English by the GHC and BRAC teams, but the Andes team also included articles written in Spanish (see Annex 1 for a more detailed methodology).

Second, the research teams held semi-structured interviews with key informants to complement the literature reviews (GHC held eight interviews, BRAC held 18, and Andes held three). The selection of interviewees varied by team, given the different purposes of the interviews. GHC interviewed experts in pharmaceutical innovation in the Global South, selected based on the authors' knowledge, to gather more information about pharmaceutical R&D activities in LMICs in general, or to understand further the innovation system in countries identified as particularly relevant in the field; BRAC interviewed key stakeholders in the pharmaceutical industry in Bangladesh, categorized into three groups: financers, implementers, and facilitators; Andes interviewed leaders from innovation accelerators, a new type of organization in the Colombian biomedical innovation ecosystem created to address translational gaps in the system (for more detailed methodology, see Annex 1).

Third, the research teams used information available in open-access databases to map, synthesize, and analyze quantitate data regarding (1) R&D funding, (2) R&D scientific capacity, and (3) clinical trial activities. For R&D scientific capacity, we used the indicator of the number of health researchers full-time-equivalent (FTE) per million habitants, published by the World Health Organization (WHO) Global Observatory on Health Research and Development in January 2022, with information on 82 countries [45]. The actual year of the data available varies from country to country.

For R&D funding, we used data on gross domestic R&D expenditure on health and medical sciences (health GERD) from the WHO Observatory (WHO Global Observatory on Health Research and Development, 2021). The data were published in December 2021, with information available for 86 countries. The year of the data differs from country to country, and the figures are from the most recent year available for each respective country. We also examined grants available for biomedical research from public and philanthropic funders in the World RePORT [46], downloaded in October 2022. The database contained information on 650,875 grants awarded to 23,005 recipient research organizations in 188 countries. It included information from 14 funders, all of whom were from high-income countries (HICs). Finally, we analyzed R&D funding for diseases “that disproportionately affect people in low- and middle-income countries” from G-FINDER based on data downloaded in October 2022, with the latest available information at the time of our analysis dating from 2020 [28].

For clinical trials, two databases were used: the WHO International Clinical Trials Registry Platform (ICTRP) [42] and ClinicalTrials.gov [35], with the latter only used by Bangladesh as of July 2022. The ICTRP consolidates information provided by several data sources, which includes 18 national and/or regional registries from around the world. The database is updated weekly and includes information dating from 1990. For our analysis, we used a cleaned data set by Merson et al. [22], containing information until 15 December 2020 on 593,595 registrations for a total of 216 countries [23]. The curated data set also included information about the income level, categorized as "high-income countries" and "low and middle-income countries" as per the World Bank classification in June 2020, and the type of sponsor, categorized as "commercial" with “evidence of profit-driven corporate mission or company structure," or "non-commercial" with “evidence of non-profit status, including governments, foundations, academic and research institutions, health care provision facilities, and public health agencies" [22, 23]. We used their curated data set, adding information about "health categories" obtained by email from the WHO Global Observatory on Health R&D. Our unique data set is available as supplementary data [19, 20].

Country case selection was shaped by the availability and eligibility for research funding and partners, as well as their relevance in the field. Bangladesh is currently classified as a lower-middle income country (LoMIC) by the World Bank and as a least developed country (LDC) by the United Nations and, as such, is exempted from implementing specific provisions of the Agreement on Trade-Related Aspects of Intellectual Property (TRIPS) of the World Trade Organization (WTO) until at least 2034, or until it graduates from the LDC status. Particularly in the pharmaceutical sector, the country is not obliged to grant patent protection and other exclusive rights. There are a few studies about the Bangladeshi pharmaceutical industry which analyze the production of generic medicines, but much less information is available about upstream R&D activities in the country. Colombia is an upper-middle-income country (UMIC) that has been very active in global debates about innovation and access to medicines, and has adopted pioneering policies in the field (e.g., regulation to accelerate access to biosimilars—[14]. It is one of the top countries in Latin America conducting pharmaceutical R&D activities, and has national policies supporting R&D activities, including public funding.

Finally, there is no single definition of “Global South.” The term has been used to refer to economically developing countries on one side of the imagined global North–South divide, which is often, but not always, geographically located in the southern part of the world. For this report, we used the World Bank classification regardless of geographical location, and used low- and middle-income countries (LMICs) as a proxy for Global South, despite the limitations of the terminology [18, 39].

## Results

### Literature reviews and interviews

This section summarizes findings from the literature reviews and interviews with key informants along four themes: (i) pathways from generic production to innovative capacity, (ii) R&D funding, (iii) actors involved in pharmaceutical R&D, and (iv) types of products and therapeutic areas. As the literature and the interviews focused on these main themes, we opted to present the results from both together, to avoid repetition.

#### Pathways from generic production to innovative capacity

Historically, developing countries have mainly focused on developing generic medicines and manufacturing, with little attention and resources allocated to innovation [10, 12, 30]. Reverse engineering of existing products was highlighted as having an important learning effect in domestic industries, thus facilitating the transition into innovative activities, referred to as an "imitation to innovation" trajectory [6, 30]. Nevertheless, the experts interviewed for the study highlighted that the current global ecosystem reduced the policy space for "imitation to innovation," as most countries are currently required to provide at least some type of intellectual property protection and market exclusivity for innovative medicines, which restricts access to information, knowledge, and technology [39].

In Bangladesh, findings showed that the national policies adopted and the TRIPS transition period for LDCs has led to the development of a local pharmaceutical industry mainly focused on developing generic medicines. This allowed the country to build a strong indigenous pharmaceutical industry, facilitated by the National Drug Policy of 1982, which aimed to ensure the supply of quality essential generic medicines at a relatively low price [17]. Private pharmaceutical companies in the country are involved with product development activities to produce generics locally for national use, and to export them to markets with different regulatory standards. However, given the costs and risks, they are still wary of investing in innovative R&D activities [17]. The pharmaceutical industry in India followed a similar trajectory—from first making generics to sell domestically, and then to export, especially to the USA and Europe, to then developing in-house R&D capacity by innovating around patented medicines, with innovation capabilities being financed by revenues from the sales of generics [3]. Local companies in Bangladesh are currently concentrating on developing reverse engineering capabilities to produce active pharmaceutical ingredients (APIs), imported mainly from China and India, which is potentially a step toward more innovative R&D [17].

The literature described different triggers for pharmaceutical innovation in LMICs. Many countries have adopted national medicines policies to secure access to low-price products, such as the Bangladesh National Drug Policy of 1982 or the Indian Patent Act of 1970 (which allowed for patents on manufacturing processes but not on final products). These policies were prompted by public health concerns that encouraged local imitation of expensive, and often unavailable imported medicines, and shaped the innovation pathway in the country, acting as a "public-policy trigger" [6], Chaudhuri, 2020). Some countries, including Brazil and South Africa, focused their policies on import substitution and lowering the cost of health products for local populations [30]. Other countries, such as India and Bangladesh, also emphasized export markets for generics, which acted as a "market-led trigger" [17, 30].

In Colombia, before the 1990s, the country followed the import substitution industrialization model, and has since started veering toward an export-led development approach [24]. Initially, science and technology were supposed to drive industrial development. However, investment in general R&D and scientific, technological, and innovation activities has been low ever since, reaching a historical maximum of 0.84% of gross domestic product (GDP) in 2020 [26, 27]. This has led to the export of low-technology products, and an accentuation of the process of de-industrialization that started in the 1990s [2]. Currently, pharmaceutical public spending is concentrated on high-cost imported pharmaceuticals, while local production of generics meets most of the demand for the most prevalent diseases. These production processes involve only packaging or fill and finish, but not the production of active pharmaceutical ingredients (APIs). Domestic manufacturers contribute 80% of the units sold in the country, but only 33% of the value, and the average price of imported medicines is eight times higher than that of those produced in the country [33].

Furthermore, the literature and interviewees highlighted several policies shaping the development of national pharmaceutical innovation systems. A few LMICs implemented policies linking R&D capacity, technological and industrial development, and public health needs, particularly in Brazil, Cuba, India, and Indonesia. Others adopted mandatory local manufacturing policies aimed at national supply security and strengthening cumulative innovation capacities, such as China and Russia. Finally, many countries require clinical trials to be conducted domestically for regulatory approval, which not only strengthens local capacity to conduct such trials, but also potentially helps to build stronger R&D capacity more generally [39].

#### R&D funding

Historically, multinational companies have concentrated their R&D activities in high-margin markets, leaving domestic companies in LMICs to address less profitable geographic and disease market segments [30]. As a result, there has been a dearth of private capital to support R&D in some LMICs, and innovative domestic companies have received significant support from governmental sources in some countries [30]. Nevertheless, a common approach for generating revenues for investment in R&D activities in LMICs, remains the manufacturing and marketing of generic medicines, in contrast with HICs, where financing for R&D often comes from capital investment [30]. Financing for R&D within firms usually comes from cash flow from the sales of generics and/or providing research services to multinational corporations, which is another strategy used by some developing countries to build up their innovation capabilities for conducting proprietary R&D [6, 9]. Many interviewees mentioned that private capital to support R&D in most LMICs was limited, and that R&D was a low political priority, leading to insufficient government policies and funding, especially beyond upper-middle-income countries (UMICs).

In Bangladesh, while universities and research organizations receive some funding from the government and other external sources, the industry is mostly self-financing. Despite generating significant revenues from the sale of generics, industry officials consider it to be insufficient to take the risk of investing in innovative R&D [17]. In Colombia, most of the R&D and scientific, technological, and innovation funding has been public, mainly from the Ministry of Science, Technology, and Innovation (Minciencias). More recently, however, there has been a reconfiguration of the funding situation in the country, with private investment surpassing public investment (e.g., in 2015, public funding for R&D in all sectors was 51%, and private was 46%, whereas in 2020, public funding was 42% and private 56%), and with international funding comprising around 2–3% of the total investment in R&D [2],OCyT report of 2020, 2021). Financial resources available to fund science, technology, and industrial activities beyond the national budget, include tax exemptions and a portion of the revenue from extractive industry activities (estimated at USD 6.7 billion for 10 years). For health-related innovation, a special fund (Fund for Health Innovation) was created with 7% of taxes collected from gambling, which fluctuates around USD 10 million per year [2].

Several key informants mentioned philanthropic funding and development assistance as important funding sources for pharmaceutical R&D in LMICs, especially in Bangladesh and Southern Africa [17, 39]. The findings also highlight the important role of international collaboration, as in many cases, R&D activities happening in the Global South are in partnership with actors outside the country or region [34, 41].

In general, there was scarce information in the identified literature about pharmaceutical R&D funding. One study mapped global investments in health R&D in 2009, and found that of the USD 240 billion spent in total, 89% (USD 214 billion) was invested in HICs and 11% (USD 26 billion) in LMICs [31]. Another study investigated global public and philanthropic funders of health research which amounted to USD 93 billion in 2013, and demonstrated that the ten largest funders (accounting for 40% of the total amount) were from North America, Europe, or Oceania [40]. Of the 55 total major funders identified by the study, 20 were based on eight LMICs (Argentina, Brazil, China, India, Mexico, Russia, South Africa, and Turkey) (ibid).

#### Actors involved in pharmaceutical R&D

The literature and interviewees frequently raised the importance of academic institutions and small and medium enterprises (SMEs), especially in the earlier stages of R&D [39]. Collaboration with domestic academic and research institutions was highlighted as an important factor contributing to private companies' R&D activities [30]. Nevertheless, the literature and interviewees highlighted that while there have been improvements [30, 37], there is still a critical translation gap between universities and industry in LMICs [2, 17, 39].

Indeed, our country-level findings identified that in Bangladesh, many academic institutions and research organizations were involved in innovative research in different fields, such as phytochemistry, ayurvedic and herbal medicine, neurology and pharmacology, and other areas to develop new drug molecules [17], while in Colombia, academic institutions also played an important role in the field [2]. However, there was a key gap in translational research, since knowledge produced at universities was usually not translated into product development, often due to weak academia-industry collaboration, lack of funding, and strategic policy direction [2, 7, 17].

The absence of targeted public policies for pharmaceutical innovation was raised as a challenge in Bangladesh and Colombia. In Bangladesh, interviewees expressed consensus regarding the need for the government to develop a favourable policy environment for pharmaceutical R&D in the country, to prepare for the end of the TRIPS transition period for LDCs. In addition, the lack of government support has been called into question, given the limited funding and support available to different actors in the field [17]. In Colombia, the literature and policy review revealed the absence of an explicit industrial policy for pharmaceuticals, but identified several dispersed policies to foster science, technology, and innovation, promote industrial production, and encourage entrepreneurship in general, as well as more general policies regulating pharmaceuticals, but not specific to pharmaceutical R&D. Interviewees mentioned that the national R&D ecosystem could benefit from a more organized public investment strategy [2].

To address the lack of cohesiveness of Colombia’s innovation system, innovation accelerators have emerged in recent years as facilitators for R&D, as well as public–private collaborations for product development in different technological sectors [2]. While public funding is instrumental in financing basic research and the early stages of product development, the interviewees highlighted the proactive role that innovation accelerators play in seeking external funding, both nationally and internationally, to support biomedical research and to give visibility to projects, which help them raise additional funds [2].

#### Type of products and therapeutic areas

Studies and interviewees suggested that, in general, pharmaceutical R&D conducted in developing countries focuses more on addressing diseases that mainly affect developing countries, addressing local needs and improving ease of use in local contexts [13, 29, 36]. As a result of developed countries outsourcing steps of pharmaceutical development to developing countries to reduce costs (such as manufacturing and clinical trials), the latter have started investing their revenues in innovating their own medicines to fill local health gaps [4]. Responding to local health needs was a key trigger and opportunity for developing countries to enter the innovation field, particularly in addressing needs neglected by multinational pharmaceutical companies [6]. One interviewee also raised necessity as an important factor driving the development of pharmaceutical R&D capacities, particularly in countries excluded or sanctioned by the international market, such as Cuba, Iran, India and South Africa (interview SI_06) [39].

In Colombia, the development of new biomedical products by both public and private research institutions or individual university researchers, mostly with public funding, was deliberately driven to address local needs [2]. Nevertheless, market returns continued to shape the R&D priorities of the private sector in LMICs, such that companies were unlikely to address diseases mostly affecting “poor market segments” [39]. For example, in Bangladesh, a few academic stakeholders mentioned that they were the only ones conducting research for some rare diseases left unattended by the private sector [17].

After decades of copying products developed elsewhere, many innovative products are being developed in LMICs [29]. A few studies and interviewees gave examples of these innovative products, indicating growing concrete outcomes from policies and investments over the years, summarized in Table 1 [39].

**Table 1:** Examples of pharmaceutical products developed in LMICs (from oldest to most recent)

Product	Type of product	Main indication	Main developer	Type of developer	Country	Years
Ultra micro analytical system (SUMA)	Diagnostics platform	Screening of several infectious diseases	Cuban Immunoassay Center	Public institute	Cuba	1986
VA-MENGOC-BC^®^	Vaccine	Meningitis B and C	National Center for Meningococcal Vaccine Development	Public institute	Cuba	1989
Ormeloxifene	Drug	Nonsteroidal oral contraceptive	Central Drug Research Institute	Public institute	India	1991
Shanvac-B	Vaccine	Hepatitis B	Shantha Biotechnics	Private company	India	1998
Abhayrab®	Vaccine	Rabies	Human Biologicals Institute National Dairy Development Board	Private company Public institute	India	2001
Gendicine^®^ (recombinant human p53 adenovirus)	Gene therapy	Head and neck cancer	Shenzhen SiBiono	Private company	China	2003
Acheflan^®^	Herbal medicine	Anti- inflammatory	Aché Laboratórios Universidade Federal de Santa Catarina Unifesp Unicamp PUC-Campinas	Private company University (public) University (public) University (public) University (private)	Brazil	2004
Nepidermin	Biologic drug	Diabetic foot ulcer	Cuban Center for Genetic Engineering and Biotechnology (CIBG)	Public institute	Cuba	2006
Nimotuzumab	Monoclonal antibody	Cancer	Centre of Molecular Immunology	Public institute	Cuba	2006
Lodenafil carbonate	Drug	Erectile dysfunction	Cristalia	Private company	Brazil	2007
CIMAvax EGF	Therapeutic vaccine	Lung cancer	Center of Molecular Immunology	Public institute	Cuba	2008
Panflu.1^®^	Vaccine	H1N1 influenza A	Sinovac Biotech	Private company	China	2009
MenAfriVac	Vaccine	Meningitis A	Serum Institute of India	Private company	India	2009
Risorine	Drug	Tuberculosis	Cadila Pharmaceutical Indian Institute of Integrative Medicine	Private company Public institute	India	2009
Icotinib	Drug	Lung cancer	Betta Pharmaceuticals	Private company	China	2011
Perchlozone	Drug	Multidrug-resistant tuberculosis (MDR-TB)	JSC Pharmasyntez	Private company	Russia	2012
Saroglitazar	Drug	Type 2 diabetes mellitus and dyslipidemia	Zydus Cadila Healthcare	Private company	India	2013
Nanoxel	Drug	Cancer	Dabur Pharma Ltd	Private company	India	2013
ROTAVAC	Vaccine	Diarrheal disease (rotavirus)	Bharat Biotech	Private company	India	2014
Bulevirtide	Drug	Hepatitis D	Hepatera	Private company	Russia	2017
Elsulfavirine	Drug	HIV	Viriom	Private company	Russia	2017
Levonadifloxacin/alalevonadifloxacin	Drug	Antibiotic	Wockhardt Ltd	Private company	India	2019
Ravidasvir	Drug	Hepatitis C	Pharco Pharmaceuticals Ministry of Health Pharmaniaga Berhad DNDi	Private company Government Private company PDP	Egypt Malaysia Malaysia Switzerland (headquarters)	2021

Source: [39]

An example from Colombia was the development of a topical cream (Anfoleish) for the control of non-complicated cutaneous leishmaniasis, which was developed by HUMAX (a Colombian private pharmaceutical company) and the Program for the Study and Control of Tropical Diseases (PECET) of the University of Antioquia, a public university, in collaboration with the Drug for Neglected Diseases initiative (DNDi), a global product development partnership (PDP), and was partially funded by the Ministry of Sciences, Technology and Innovation (Minciencias) [5, 11, 21].

In Bangladesh, despite the translational gap discussed above, one of the successful examples of pharma–academia collaboration was the development of a herbal medicine for cough relief (Adovas^®^ syrup) by a top pharmaceutical company (Square Pharmaceuticals), through research carried out by a top university according to a key informant [17]. It is also worth mentioning that a private company in Bangladesh (Globe Biotech Limited) started developing its own COVID-19 vaccine candidate (Bongovax) during the pandemic, indicative of more innovative R&D activities [17].

In general, there is less pharmaceutical R&D happening in the Global South compared to the Global North, usually attributed to a lack of funding, R&D facilities and infrastructure, and human resources [29]. Research in the pharmaceutical sector is highly concentrated in HICs, encompassing nearly 72% of all research conducted [16]. Nevertheless, the literature and interviews showed growing pharmaceutical R&D activities in LMICs, with growing outputs. The development of the pharmaceutical industry is, however, uneven among countries in the Global South, with a few in the lead and growing at considerable speed. Indeed, most of the studies identified in the literature search referred to only a few LMICs, namely, China, India, Brazil, South Africa, and Cuba. These findings from the literature are largely consistent with this study’s analysis of data from interviews and databases, as described further in the following sections.

### Indicators of pharmaceutical R&D capacities and activities

To complement the literature and interview findings, we analyzed information from publicly available databases on different aspects of pharmaceutical R&D, as specified in the methodology section. The databases provided insight into pharmaceutical innovation capacities and activities in LMICs. Still, they were limited in scope in terms of diseases, stages of R&D covered, and countries and funders providing data. Below, we present an analysis of the available information on (1) R&D funding, (2) R&D scientific capacity, and (3) clinical trial activities. For some indicators, LMICs are further divided into three categories: upper-middle income countries (UMICs), lower-middle income countries (LoMICs) and low-income countries (LICs).

#### R&D funding

The WHO Global Observatory on Health Research and Development provides global data that allows health R&D activities to be monitored, building on existing data and reports from various sources and WHO member states' reports [45]. The available data are not comprehensive and is often related to only a few diseases (in particular, neglected diseases). Still, it is representative of the data sources available on the topic and can provide valuable information regarding, where pharmaceutical R&D activities are taking place, for what, and by whom.

**Fig. 1:**
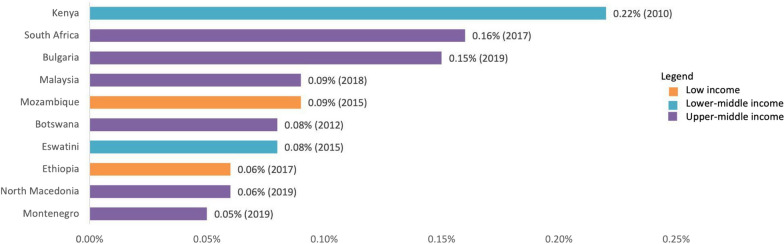
Top 10 LMICs by % of GDP invested in health GERD. [43] Source: Data from the WHO Global Observatory on Health R&D

For R&D funding, we used the data on gross domestic R&D expenditure on health and medical sciences (health GERD) from the WHO Observatory (WHO Global Observatory on Health R&D, 2021a). South Africa had the highest percentage of GDP invested in health GERD among UMICs (0.16%, 2017), Kenya among LoMICs (0.22%, 2010), and Mozambique among LICs (0.09%, 2015) (Fig. 1). There was no data available for Bangladesh or Colombia in the database. The data available was not suitable for analysis of the variation over time, as most countries did not report information for more than 1 year in the period covered by the Observatory.

We then examined grants awarded for biomedical research from public and philanthropic funders in the World RePORT [46]. At the time of our analysis, the database contained information on 650,875 grants awarded to 23,005 recipient research organizations in 188 countries. Among LMICs, South Africa received the highest number of grants (7,044), followed by China (4,851), and Kenya (3,553). Bangladesh received 796 grants, and Colombia received 583. Regarding the number of research organizations who received grants, 74% were based on HICs, while 12% were based on UMICs, 10% in LoMICs, and 4% in LICs. Among upper-MICs (UMICs), China (571), South Africa (500), and Brazil (346) had the highest number of research organizations who received grants; among lower-MICs (LoMICs), it was India (476) and Kenya (304), while among low-income countries (LICs), it was Uganda (247) and Malawi (110). Colombia had 100 research organizations who received grants in the database, while Bangladesh had 52 (Table 2).

**Table 2:** Top* LMICs by number of research grants received, World Report, 2022

Country/Income group	Number of grants received	Number of research organizations
South Africa (UMIC)	7,044	500
China (UMIC)	4,851	571
Kenya (LoMIC)	3,553	304
Uganda (LIC)	3,458	247
India (LoMIC)	3,133	476
Brazil (UMIC)	3,026	346
Malawi (LIC)	1,166	110
Bangladesh (LoMIC)*	796	52
Colombia (UMIC)*	583	100

Source: Data from [46]

*Bangladesh and Colombia are not among the top countries in their respective income groups, but, as focus countries, were added to the table for comparison

We then analyzed R&D funding for diseases “that disproportionately affect people in low- and middle-income countries” from G-FINDER [28], using the data for funding that was awarded and received (Table 3). The total funding tracked in the period from 2007 to 2020 amounted to approximately USD 61.5 billion. Of this funding, 81% came from HICs, 2% from LMICs, 1% from UMICs, and less than 1% from LICs, with the remaining funding unclassified. From 2010 to 2020, there was an increase of more than 450% in the total amount funded by middle-income countries (MICs), while funding from LICs remained roughly the same. India (USD 921 million) was the most significant public funder among LMICs. Colombia funded USD 36 million during the time period, and there were no records for Bangladesh as a funder.

**Table 3:** Amount funded and received for health R&D by income group and country, G-FINDER, 2007–2020

Income group/Country	Amount funded (USD)	Amount received (USD)
High income	49,514,838,988	40,849,557,815
Low income	520,803	194,517,036
Rwanda	250,124	155,766
Gambia	112,869	40,386,664
Ethiopia	97, 273	8,022,652
Malawi	0	66,878,265
Uganda	26,993	40,228,555
Lower middle income	931,455,043	1,236,080,823
India	921,038,235	960,557,650
Egypt	4,642,225	4,999,887
Indonesia	2,178,064	3,785,469
Ghana	36,884	80,936,027
Bangladesh	0	51,355,381
Kenya	152,415	48,922,220
Upper middle income	527,299,949	1,150,535,609
Brazil	228,032,716	253,008,026
South Africa	111,831,640	486,540,102
Russia	41,567,705	42,587,120
Colombia	36,100,113	55,066,738
China	27,030,281	87,306,008
Unclassified	10,523,440,761	18,066,864,262
Grand total	61,497,555,545	61,497,555,545

Source: Data from [28]

Regarding funding received during the same period, HICs accounted for over 66% of the funding, while UMICs and LMICs accounted for about 2% each, and LICs accounted for only 0.3%, with the remaining funding unclassified. Funding received by LMICs also increased over time, indicating a growing capacity for conducting R&D. In 2010, LICs received USD 13 million, and MICs received USD 162 million. In 2020, LICs received USD 16 million, and MICs received USD 250 million. India (USD 960.5 million) and South Africa (USD 486.5 million) were the top receiving countries among LMICs. Meanwhile, Colombia received a total of USD 55 million during the period, while Bangladesh received USD 51.3 million.

#### R&D scientific capacity

**Fig. 2:**
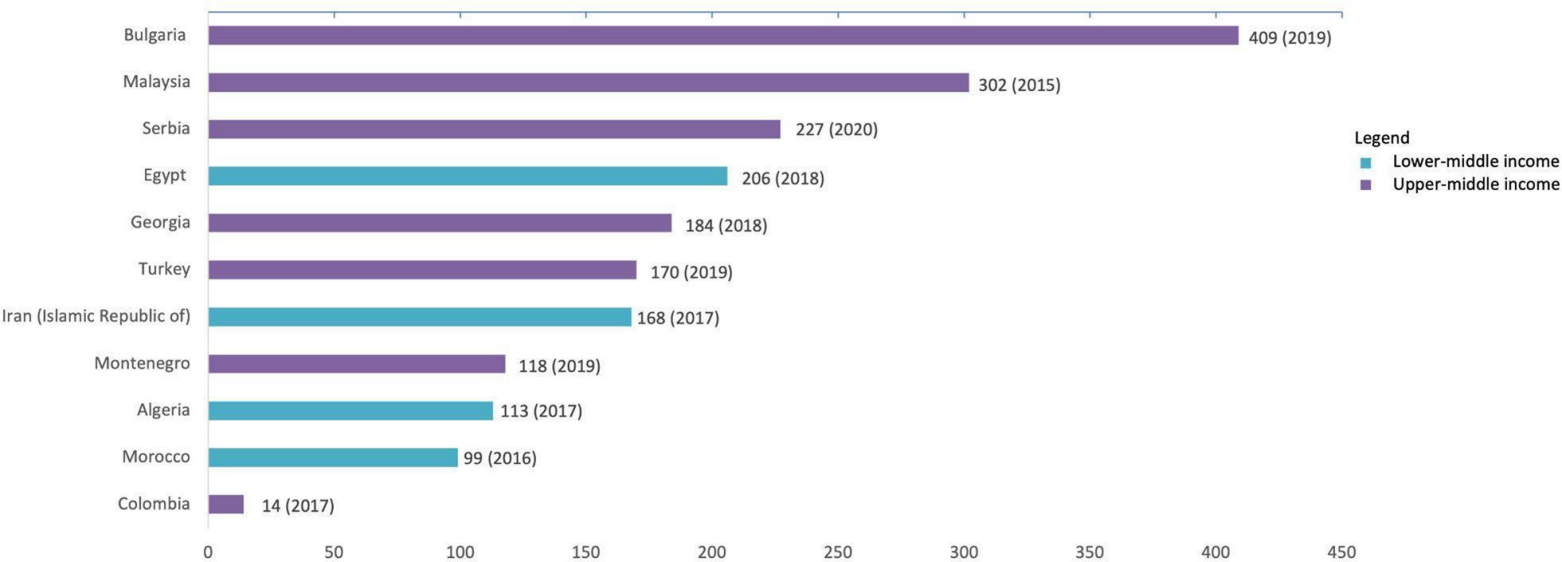
Health researchers per million inhabitants by country in the top* LMICs, 2021. [44]. *Colombia was not among the top LMICs, but, as a focus country, was added to the figure for comparison Source: Data from WHO Global Observatory on Health R&D

For R&D capacities, we complemented the analysis using the indicator of the number of health researchers full-time-equivalent (FTE) per million habitants from the WHO Observatory [44]. Bulgaria had the highest number of health researchers among UMICs (409 FTE, 2019), Egypt among LoMICs (206 FTE, 2018), and there were no LICs among the top 10 LMICs. Colombia was in 29th position amongst LMICs, with 14 FTE health researchers per million habitants (Fig. 2). For comparison, the average in the UMICs income group was 107 FTE. There was no data available for Bangladesh for this indicator. Again, the data were not suitable for a comparison over time.

#### Clinical trials

We then analyzed clinical trial activities by looking at the number of trials, phases, types of diseases, sponsors/funders involved, and variation over time.

##### Number of trials

**Fig. 3:**
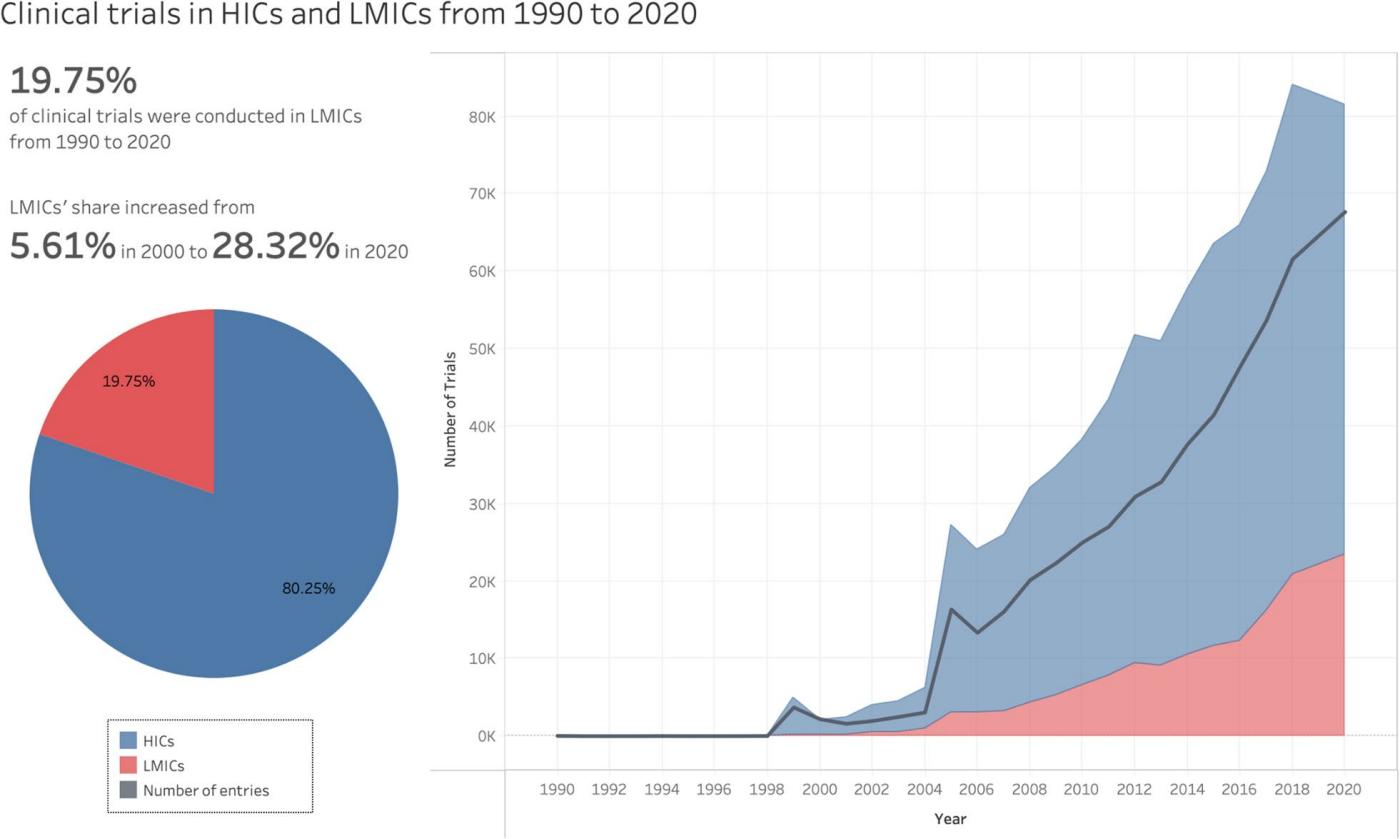
Number of clinical trials by income level (1990–2020). [39] Source: Vieira et al.

During the period covered (1990–2020), most clinical trials were conducted in HICs (690,963; 80%), but growth in LMICs was rapid, particularly in China, India, and Iran. The number of trials increased from 6,498 in 2010 to 22,960 in 2020, an increase of 375% in the period (Fig. 3).

**Fig. 4:**
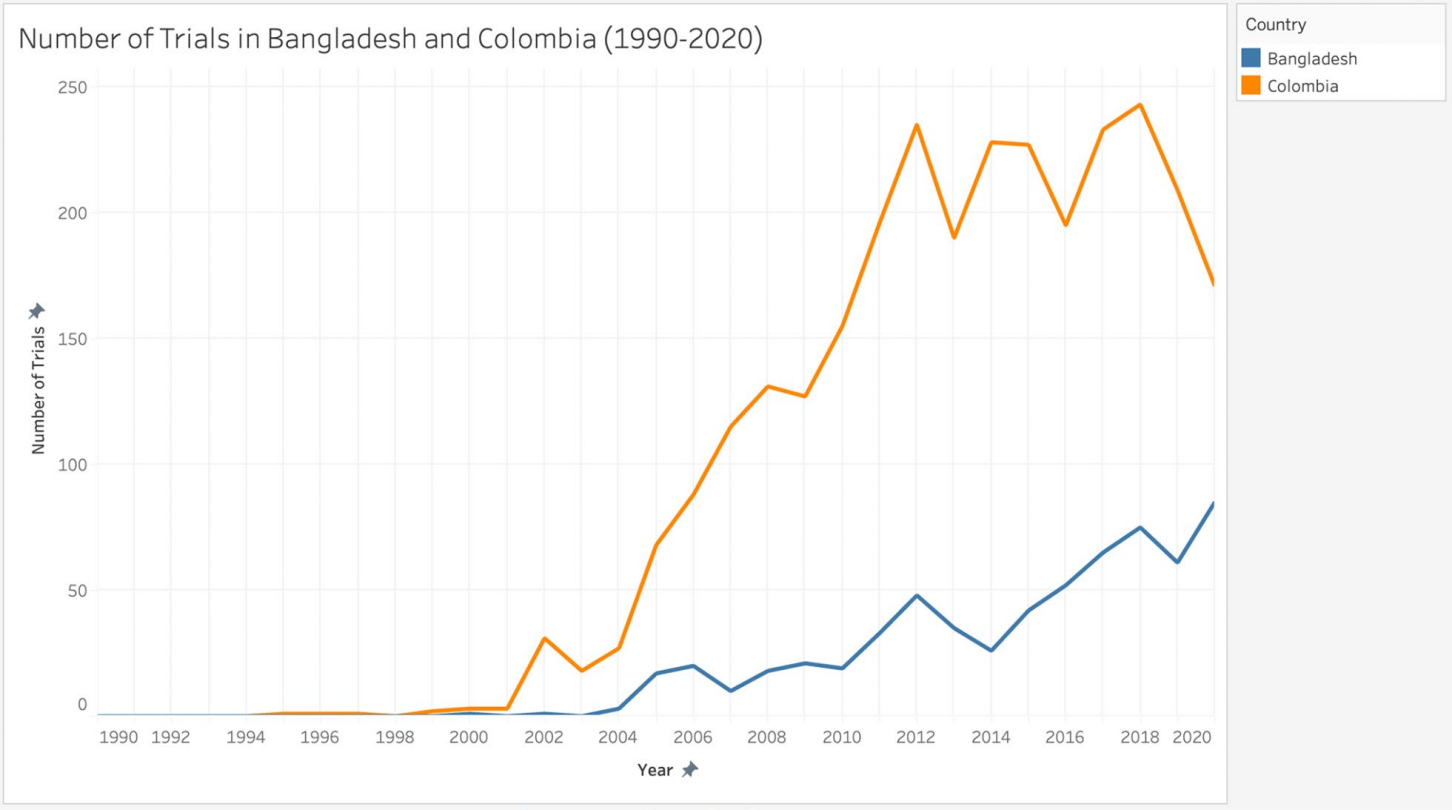
Number of clinical trials in Bangladesh and Colombia by year (1990–2020). Source: Data from [22]

The data set included 632 clinical trials in Bangladesh and 2,898 in Colombia. To analyze the variation over time, the graph below shows the number of trials in Bangladesh and Colombia by year from 1990 to 2020. From 2010 to 2020, there was a 347% increase in the total number of trials in Bangladesh, from 19 in 2010 to 85 in 2020. In Colombia, the increase was 10%, from 155 in 2010 to 171 in 2020 (Fig. 4).

##### Phases

Furthering the analysis, we sought to identify which phases of clinical trials were most frequent in LMICs. In total, 56% of the entries (334,918) did not have information on phases. Of those with information available, most trials were in Phase 3, both in HICs and LMICs. However, there was a growing number in Phase 0 in LMICs. Notably, China and India had a large proportion of trials in Phases 0 and 1, and Egypt and Thailand showed significant growth in earlier phases from 2010 to 2020. These trends suggest increasing capacity in the riskier, more innovative, earlier stages of R&D.

Fifty-eight percent of clinical trials conducted in Bangladesh had no information on the phase. For 42% of trials that did have this information available, the majority of trials were Phase 2 (54), Phase 3 (77), and Phase 4 (62), while only 3.6% of trials were registered as Phase 0 (2) and Phase 1 (21). In Colombia, about 80% of the trials had information available on the phase, and the majority of trials were Phase 3 (1,637; 56%) and Phase 2 (377; 13%). Phase 0 (6) and Phase 1 (37) represented only 1.5% of the trials in the country. In Bangladesh, from 2010 to 2020, there was an increase in trials in Phase 1/2 (400%) and Phase 3 (200%), and a decrease in trials in Phase 4 (−75%). In Colombia, there was most significantly an increase in Phase 1/2 (100%) and a decrease in Phase 4 trials (−91%). Phases 0 and 1 trials remained roughly the same; still, the increase in Phase 1/2 in both countries can be indicative of more R&D activity in the early stages of clinical development, which are usually more risky and innovative.

##### Types of diseases

We also analyzed the disease category of the trials, using the categorization obtained by email from the WHO Global Observatory on Health R&D which was added to our data set. For the analysis, we used the information categorized into 27 different health sub-categories, including "unknown" (hereafter referred to as "disease category"). The disease category with the largest number of trials was malignant neoplasms both in HICs (22%) and LMICs (11%), while infectious and parasitic diseases represented about 5% of trials in HICs, and 9% in LMICs. Over time, there was a significant increase in trials for respiratory diseases in all countries from 2010 to 2020 (1,104% in HICs and 2,146% in LMICs). In LMICs, other categories also increased significantly, particularly congenital anomalies (1,417%) and oral conditions (1,042%).

In Bangladesh, “infectious and parasitic diseases” had the highest number of trials (173; 28%), while in Colombia, it was “malignant neoplasms” (465; 17%). In Colombia, sponsors of clinical trials were classified into national and international (see the section below), demonstrating that international sponsors were more involved with research for "malignant neoplasms" (457; 17,5%), while national sponsors were more involved with "cardiovascular diseases" (33; 11%). Comparing the numbers from 2010 and 2020, in Bangladesh, the highest increases were in the number of trials in “respiratory diseases” (2,600%) and “neuropsychiatric conditions” (1,100%). In Colombia, the highest increases were in “respiratory infections” (583%) and “ill-defined injuries/accidents” (300%).

##### Sponsors and funders

In addition, we analyzed the number of trials according to the sponsor/funder type,^2^ as categorized in the curated data set by Merson et al. [22]. Primary sponsors are the main sponsors responsible for the trial, while secondary sponsors assume responsibilities agreed upon with the primary sponsors. Funders are major sources of financial support for the trial. The primary and secondary sponsors, as well as funders, were classified as either commercial or non-commercial (see methodology for definitions). The categorization of the funders was available for about 35% of the trials (210,547). Almost all trials (99%, 589,373) had information on the primary sponsor, and only 24% (142,379) of the trials had information on the secondary sponsor. For Bangladesh and Colombia, almost all trials had information about primary sponsors, and 47% and 17% had information about secondary sponsors, respectively. With respect to funders, information was available for about 33% of the trials in Bangladesh and about 36% in Colombia.

**Fig. 5:**
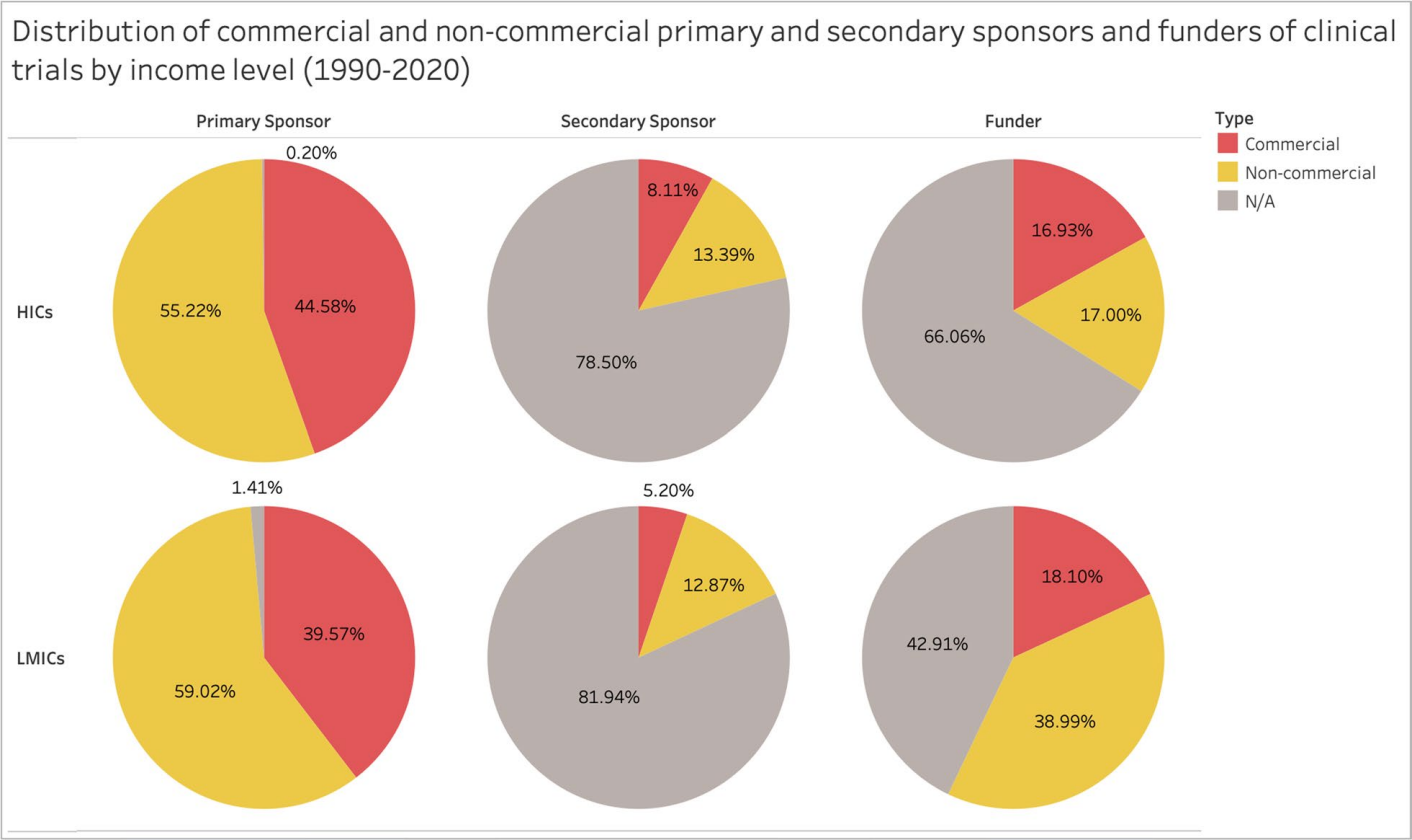
Distribution of commercial and non-commercial sponsors and funders by income level (1990–2020). [39] Source: Vieira et al.

The analysis of trial sponsors and funders showed a higher number of non-commercial than commercial sponsors in HICs and LMICs, and non-commercial funders in LMICs (Fig. 5). Moreover, we observed increasing involvement of non-commercial sponsors and funders over the past years, particularly in the early R&D phases. In some areas, such as maternal conditions, sexual health, perinatal conditions, and nutritional deficiency, non-commercial actors played a markedly dominant role. In Bangladesh, non-commercial primary (85%) and secondary sponsors (41%) were prevalent, while in Colombia, there were many more commercial primary sponsors (82%) and about the same distribution of secondary sponsors (roughly 8.5% each). In Bangladesh, more trials were funded by non-commercial (25%) than by commercial funders (6.5%), while in Colombia, there were more trials funded by commercial funders (33%) than by non-commercial (4%).

Going deeper into the analysis of the actors involved in clinical trial activities, in Colombia, the primary sponsors were classified into national and international. From the 2,898 clinical trials in the data set, about 90% of the trials were sponsored by international organizations (2,597 records), while national sponsors participated in approximately 10% (301 records). From 2010 onwards, there has been a continuous growth in the number of clinical trials conducted by national sponsors, going from 14 (9%) in 2010 to 46 (27%) in 2020. Regarding the commercial vs. non-commercial categorization, it was found that about 91% of the international sponsors were commercial, while less than 9% were non-commercial. For the national sponsors, 4% were commercial, while 96% were non-commercial, which is in complete contrast to the international sponsors.

**Fig. 6:**
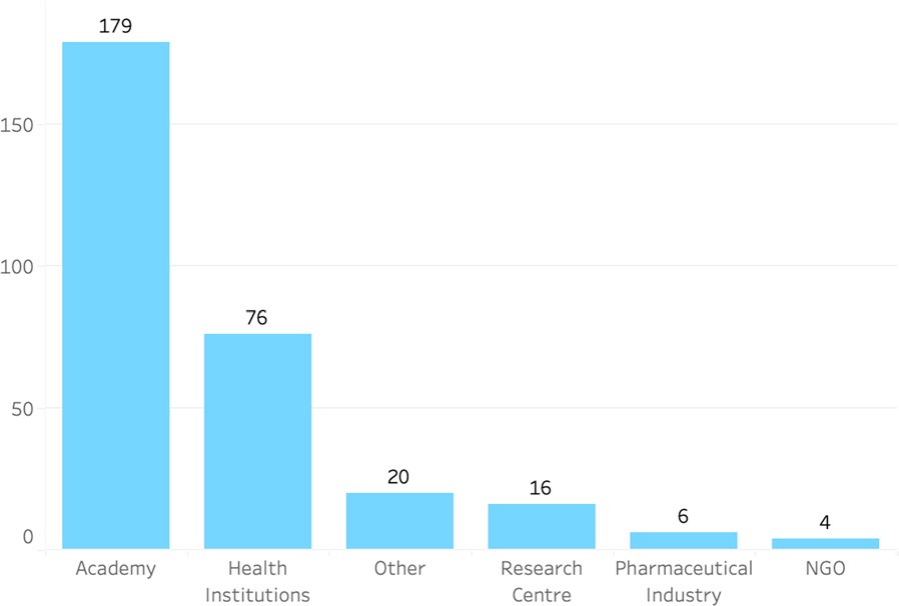
Categories of national organizations that are primary sponsors, Colombia. Source: [2]

Furthermore, national sponsors were categorized into academic institutions, research centers, health institutions, non-governmental organizations (NGOs), pharmaceutical companies, or other types of institutions. Academia (public and private universities) was the largest group (179), followed by health institutions (76), others (20), research centers (16), pharmaceutical companies (6), and NGOs (4) (Fig. 6).

**Fig. 7:**
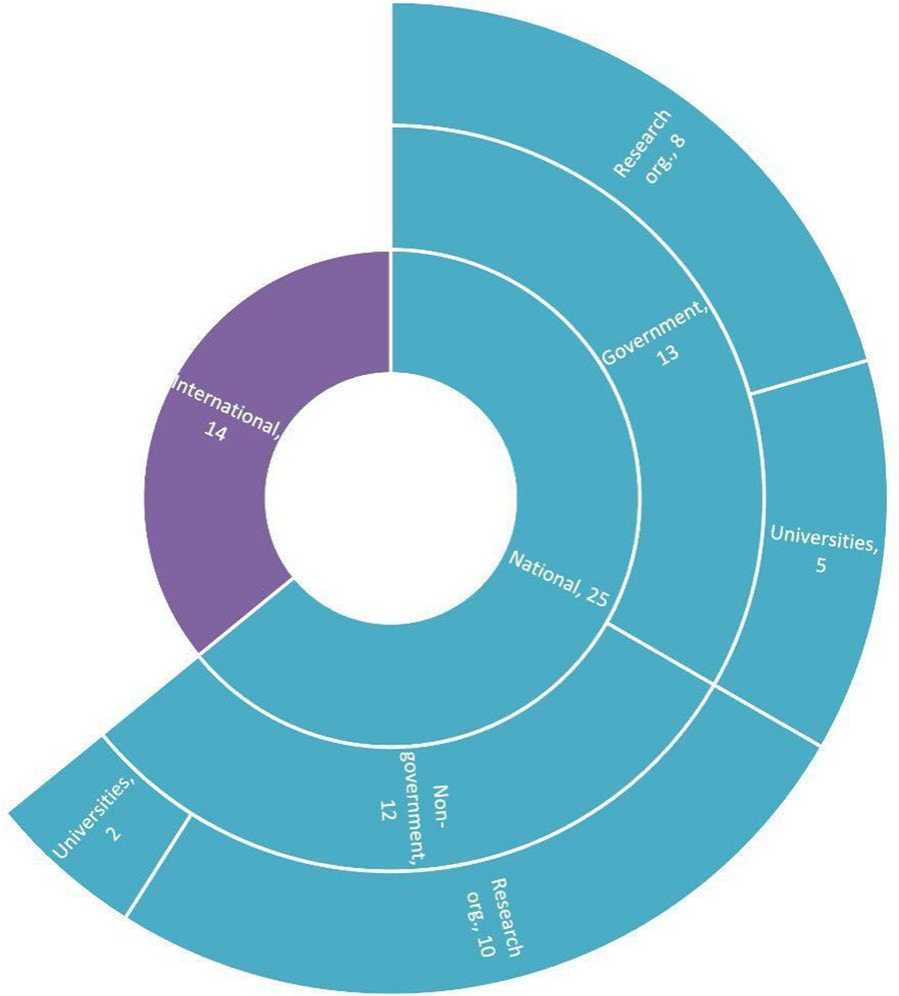
Classification of organizations involved in clinical trials in Bangladesh. Source: Data from [17]

The BRAC team analyzed information obtained from the database ClinicalTrials.gov, categorizing the organizations into international and national organizations, and further categorizing national organizations into government and non-government research institutes and universities. As of July 2022, the database contained information for 500 clinical trials conducted in Bangladesh. Thirty-nine organizations were involved in 337 clinical trials in the country, from which eight were national government research organizations and five were government universities; ten were non-government research organizations and two were non-government universities; and 14 were international organizations, with the remaining unclassified [17]—refer to the full research report for the list of organizations). National non-government organizations conducted the highest number of clinical trials (91,38.2%), followed by national government organizations (115; 23%) and international organizations (31; 6.2%) (Fig. 7).

## Discussion

Notwithstanding the growing importance of, and interest in, pharmaceutical R&D capacities and activities in the Global South, there is relatively limited information available in the public domain. Despite existing data limitations, by triangulating data from the literature, interviews, and publicly available databases, it was possible to paint a picture of who is involved in pharmaceutical R&D in LMICs, in which particular countries, for which diseases, in which R&D phases, and with what results—as well as how these trends have changed over time.

As shown by the indicators analysed, i nvestment in health R&D has increased in LMICs in the past decade, particularly from MICs. Capacity has also grown, with an increase in the number of research organizations and the amount of funding received from external sources. Not only has the total number of trials and the proportion of all trials in LMICs increased, but there is also growing activity in the earlier, more innovative and riskier phases. Finally, investments in building R&D capacities have already borne fruit, as indicated by several health technologies developed in LMICs, as seen above in the findings from the literature and interviews. The list of products developed in the Global South can be expected to grow in the coming years.

A number of LMICs were highlighted in the literature as conducting important pharmaceutical R&D activities, particularly China, India, Brazil, South Africa, and Cuba. The analyzed data showcased countries with the highest investments and capacities in R&D, based on the following indicators: percentage of GDP invested in health GERD, number of health researchers per million inhabitants, number of recipient research organizations and research grants received (World RePORT), amount funded and received for health R&D (G-FINDER), and number of clinical trials. Table 4 provides a list (in alphabetical order) of the top countries most often mentioned in pharmaceutical R&D, combining information from each source analyzed throughout the research.^3^

**Table 4:** List of top LMICs in pharmaceutical R&D combining different sources (in alphabetical order)

Top 16 LMICs involved in pharmaceutical R&D			
Brazil	Cuba	Iran	Russia
Bulgaria	Egypt	Kenya	Serbia
China	Georgia	Malaysia	South Africa
Colombia	India	Mozambique	Uganda

Source: [39]

While an analysis of the factors that make these particular countries lead the ranking of pharmaceutical innovation in the Global South is beyond the scope of this study, the literature, interviews, and the country’s case studies, show the importance of national regulations and political prioritization of the area. Both in Bangladesh and Colombia, the lack of a targeted innovation policy in the pharmaceutical sector was raised as an important challenge for strengthening pharmaceutical R&D in the country.

In Colombia, the findings showed that while the government plays an important role in funding basic research, mostly in academic and research institutions, it has been insufficient to guarantee the advancement of biomedical research into later stages of development. More recently, government funding has turned towards applied research, which is perceived as a way of addressing the translational gap, but is taking funding away from basic research and discovery, foundational and riskier phases of the R&D process. In addition, there is no centralized data source of the innovation taking place in the country or how it could be used to address local needs, and it is challenging to track the history of projects funded with public resources [2]. Innovation accelerators, both public and private, have emerged in the country seeking to address the translational gap by bridging research and production, and also by bridging the funding gap, receiving funding from both the national government and external sources. Nevertheless, the research concluded that there is a need for the national government to play a more proactive role in building bridges between the different actors involved in pharmaceutical R&D in the country [2].

In Bangladesh, the findings also revealed that the role of the national government was perceived as being very limited, with some government funding available for basic research for pharmaceutical R&D in academia, but no direct support to the private sector [17]. The current business model of the pharmaceutical industry in the country can be summarized as profit and market-driven, with private pharmaceutical companies manufacturing generic formulations of medicines developed abroad, to generate revenues from sales in the domestic and international markets, but with very little being invested in R&D for the development of innovative medicines [17]. In conclusion, urgent attention and investments, both from the government and the private industry, are needed to prepare the country for sustainability in the sector once it is no longer eligible for the TRIPS LDC transition period [17].

In both countries, different stakeholders recommended developing targeted policies for pharmaceutical R&D, with better coordination between the various parts of the domestic innovation system to take product development to the end of the process. In Bangladesh, it was suggested that the government should have a dedicated budget for the sector while also adopting incentives to de-risk investments in pharmaceutical R&D in the private sector, such as the deduction of corporate income tax, insurance policies, or exempting R&D expenses from taxes. Meanwhile, there could be a specific requirement for private companies to invest a percentage of their profits in innovative R&D. It was also raised that Bangladesh should negotiate with its development partners to secure financial and technical assistance and technology transfer [32]. These actions should encourage more investment in pharmaceutical R&D, moving from an imitative to an innovative strategy for the sector [17].

## Conclusions

Pharmaceutical R&D activities are happening in a wide range of LMICs, but 16 countries have emerged as frontrunners in the indicators analyzed in our research. While a deeper understanding of the pharmaceutical innovation systems of each country was beyond the scope of this research, the literature and interviews, as well as the country studies, highlighted the importance of national regulations, policies, and laws to shape the development of national pharmaceutical innovation systems.

While the findings from our research collaboration provided a baseline snapshot, ongoing systematic data collection and analysis of R&D activities in LMICs is still needed. Country-level studies beyond Bangladesh and Colombia, analyzing strengths, weaknesses, and trajectories, are also needed to deepen understanding of effective policies for building R&D capacity. Pharmaceutical innovation in the Global South is a rich, promising, and rapidly evolving area with strategic importance for global health, which merits far more research and attention than it has received to date.

Finally, the research showed that non-commercial actors play a significant and growing role in clinical trials, especially in LMICs. In Bangladesh, the vast majority of sponsors and funders of clinical trials are of non-commercial nature. In Colombia, while 90% of international sponsors are commercial, almost all national sponsors are non-commercial. The important role of non-commercial funders and sponsors, and the disproportionate increase in the proportion of trials they supported over the past decade, suggests they play a much more significant role in R&D than is widely understood. The high number of non-commercial actors in LMIC R&D suggests that there is fertile soil to experiment with alternative R&D models that are not driven primarily by market incentives.

## Figures and Tables

**Fig. 8:**
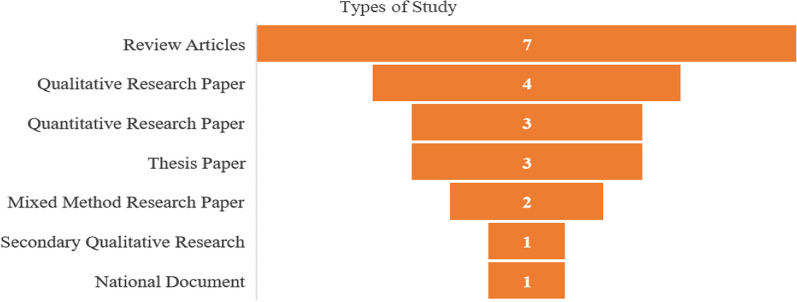
Types of study used for data extraction and analysis (BRAC)

## Data Availability

Three research reports with data supporting this manuscript are available at https://www.knowledgeportalia.org/resources. Two files with supplementary data used for the analysis of clinical trials are available at: https://zenodo.org/record/7801929 and https://zenodo.org/record/7802113.

## Appendix

### Methodology

The study followed a three-step methodology for the analysis of pharmaceutical research and development in the Global South: (1) literature review, (2) semi-structured interviews with key informants, and (3) quantitative database analysis. Below we provide more detailed information about the methodology followed by each research team for the literature review and interviews, which are also available in the full research reports.

#### Literature review

The literature review aimed to provide a broad overview of the literature, and was not intended to be a systematic literature review of the topic. Each research team followed a similar methodology, with variations, as detailed below.

Andes: conducted a policy and literature review on biomedical innovation in Colombia. Searches were conducted both in English and Spanish with keywords in major databases, especially PubMed, SciELO, ScienceDirect, Scielo, Wiley, from the earliest available literature until April 2022. Keywords include “pharmaceutical”, “drug”, “medicine”, “vaccine”, "health", “innovation”, “research and development”, “product development”, “biomedical innovation”, “Colombia”. Reports from national government, international and regional organizations, comparative documents in which Colombia is a case study, and consulting firms, are also included. The literature review includes papers and reports in Spanish and English, therefore, subject to limitations.

BRAC: scoping review (ScR) for secondary data analysis: to explore the current situation of Pharmaceutical R&D, a scoping review of available documents was done. We used Google, Google Scholar, PubMed, Scopus and Research4life for searching relevant literature from Bangladesh. Ultimately, 21 articles were included for analysis of which 12 were primary research papers, eight were secondary review papers and one was a national document. Of these, seven are review articles, one secondary qualitative research paper, four qualitative research paper, three quantitative research paper, two mixed method research paper, three thesis paper and one national document. Moreover, 3 of the articles dealt with the research expenditures and pricing of the R&D, 3 articles were about the prospects and growth of pharmaceutical industry in Bangladesh, 4 articles focused on the innovative capacity of pharmaceutical R&D, 6 articles dealt with TRIPS and IPR related implications & challenges, 2 articles related to marketing and management practices, one document on the history of pharmaceutical evolution and one national budget document were included (Fig. 8).

GHC: the literature on pharmaceutical research and development (R&D) provides information mostly about activities in the Global North, especially in the United States and Western Europe, the two most significant contributors to innovative products (which in particular include Germany, France, the United Kingdom, and Switzerland) (IDEA Pharma 2022; [1, 15, 29]. There was limited information on pharmaceutical R&D in the Global South, including where R&D activities are conducted, by whom, what products have been developed or are under development, and what policies or regulations are in place. To help fill this knowledge gap, a literature review which focused on countries in the Global South was conducted.

Searches were conducted in English in major databases, including PubMed, SciELO, and Global Index Medicus, from the earliest available literature until April 2022. Keywords and search terms included “pharmaceutical”, “drug”, “medicine”, “vaccine”, "health", “innovation”, “research and development”, “product development”, “global south”, “developing countr*”, “emerging countr*”, and “low–middle-income countr*”. To complement the search, we manually snowballed references in the selected articles and used the tool Litmaps to trace citations of key articles (in October 2022). Grey literature, including reports from national governments, international and regional organizations, and consulting firms was also included through targeted research on Google, primarily to complement information not available in the identified academic literature. Due to resource constraints, we did not conduct literature searches on each developing country or region, and this is a significant limitation of the review, as is the limitation of the search to only English language sources. Nevertheless, we believe this is the most comprehensive recent literature review on pharmaceutical innovation in the Global South publicly available in English.

#### Interviews with key informants

To complement the literature review, each team conducted semi-structured interviews with key informants, following the methodology detailed below. Sample interview questions are available in the full research reports.

Andes: interviewed leaders from innovation accelerators to gather information on how the biomedical innovation ecosystem works in practice in Colombia. Given that prior research focuses mainly on the perspectives of policy-makers and/or researchers, we decided to focus on this new set of actors that act as brokers between research and production and that give a fresh perspective of how R&D investments can become actual accessible products. The interviewees were selected based on the internal knowledge of the research team and followed up by snowballing. The selected individuals are from private and public organizations with extensive experience in knowledge transfer, pharmaceutical policy, access to medical devices and industry-researcher networking. These are the innovation accelerators interviewed:

1. Ruta N: innovation and Business Center of the city of Medellín. This public corporation was created by the local government, and Public Enterprises of Medellín (EPM) a state-owned organization, to promote the development of technology-based businesses to increase competitiveness in the city. We find this initiative interesting, because it has three specific focuses, health technologies being one of the most relevant areas.
2. Tech Innovation Group (TIG): organization focused on the development of companies dedicated to technologies that contribute to human, animal, and environmental health. The organization seeks to solve unmet needs under the principles of conscious capitalism, economic development, and sustainability. We are particularly interested in this organization's diverse approach to health that includes animals, humans, and the environment.
3. PECET—Programa de estudio y control de enfermedades tropicales: Universidad de Antioquia’s multidisciplinary research group in tropical diseases. Founded in 1986 by Ivan Darío Vélez, it started by researching Leishmaniasis and has further expanded its scope and capacity. With over 250 research projects and 340 articles, PECET has developed their work in Colombia, Central America, Africa, The Mediterranean, and Asia. PECET has received significant funding from MinCiencias and has partnered with domestic and multinational industries and pharmaceutical companies.

On top of the innovation accelerators we interviewed there are two additional innovation accelerators we were unable to interview because of the transition between governments at the time of the research and the difficulty contacting leaders of these institutions. These are:

4. Fastrack Institute: Medellin-based Fastrack Institute (FTI) is a non-profit organization co-founded with Salim Ismail, former Innovation Director at Yahoo, co-founder of Singularity University and with Maurice R. Ferré MD, co-founder, and former CEO of Mako Surgical, current CEO of Insightech. According to their webpage, they are a “non-profit organization that accelerates technology into society by finding holistic approaches to solving problems, with a focus on large urban centers.” Also, according to them, to achieve their objectives they use mobility, health, financial inclusion, and air quality as their key areas of focus.
5. Innpulsa: public agency for entrepreneurship and innovation of the Colombian government. It is aimed at ventures throughout the national territory, with projects of high social sense and innovative ideas in a wide variety of topics related to productive sectors relevant to the country. This initiative is one of the largest in the country. They are interested in understanding how a national public organization supports entrepreneurship and research in health and its relationship with public and private organizations.

BRAC: Qualitative Key-Informant Interviews (KIIs) with key stakeholders: for a comprehensive understanding of the pharmaceutical R&D, their business model, exploring the possibility of adopting alternative business models for their pharmaceutical R&D, KIIs were considered appropriate. 18 KIIs were from different background. The respondents had been categorized into three groups: financers, Implementers, and facilitators. Financiers are authorities of the pharmaceutical companies responsible for deciding on R&D funding strategy, allocation and recipients. We specifically targeted executive directors, managing directors, or general managers, since, in the context of our country, they are the ones who decide how much money should be allocated to different departments within the company, including R&D. Second, the group of people who are actively involved in R&D is known as implementers, and we contacted the head of R&D when looking for implementers. Last but not least, facilitators, including policymakers, intergovernmental organizations, data-sharing platforms, patent pools, product development collaborations, matching initiatives, and analysts, are the ones shaping R&D. We interviewed eight facilitators from various universities and governmental agencies, as well as four financiers and six implementers from well-known pharmaceutical companies (from top 20 companies). All of them are top professionals having more than 15 years of experience. The respondents were categorized into three (3) groups: implementers, financiers and facilitators. The data were collected from different pharmaceutical companies (Top, medium and small companies), academicians and policymakers. For KIIs, we used a combination of purposive and snowball sampling to identify and reach relevant stakeholders. We conducted a total 18 KIIs (Table 5).

**Table 5:** Study population and sample size (BRAC)

Categories	Key informants participated	Numbers of KIIs
Implementors	Head of Research/Research Unit Directors, Quality control/assurance officers	6
Financers	MD/CEO of the pharmaceuticals with R&D or Product Development	4
Facilitators	DGDA, academics/faculties/researchers (pharmacy & pharmacology) of different universities and research organizations	8

GHC: to complement the literature, interviews were conducted early in the project with experts in the field of pharmaceutical R&D in the Global South. Interviewees were selected based on the authors' knowledge and aimed for geographical representation. In total, 12 people/organizations were contacted for interviews, and seven interviews were conducted with eight individuals (58% response rate). The interviews aimed to gather more information about pharmaceutical R&D activities in LMICs in general, or to understand further the innovation system in countries identified as particularly relevant in the field, especially Brazil, Russia, India, China and South Africa (BRICS). Information from the interviews has been anonymized. Sample interview questions are available at the full research report.

List of interviewees (in alphabetical order of last names)

- Michelle Childs, Drugs for Neglected Diseases Initiative (DNDi)
- Gabriela Costa Chaves, Independent researcher (licensed from Fiocruz)
- Spring Gombe, former consultant at United Nations Development Programme (UNDP)
- Lynette Mabote, Sustainable Access to Pharmaceuticals & Affordability Models (SAPAM)
- Achal Prabhala, Access IBSA—India, Brazil and South Africa
- Judit Rius Sanjuan, United Nations Development Programme (UNDP)
- Robert Terry, TDR—Special Programme for Research and Training in Tropical Diseases
- Anonymous, civil society organization, Russia

## Abbreviations

APIs: Active pharmaceutical ingredients
FTE: Full-time equivalent
Health GERD: Gross Domestic R&D Expenditure on health and medical sciences
GDP: Gross domestic products
GHC: Global Health Centre
ICTRP: International Clinical Trials Registry Platform
IFPMA: International Federation of Pharmaceutical Manufacturers and Associations
LDC: Least developed country
LICs: Low-income countries
LMICs: Low- and middle-income countries
LoMICs: Lower middle income countries
MICs: Middle-income countries
NGOs: Non-governmental organizations
OCyT: Observatorio Colombiano de Ciencia y Tecnología
PDP: Product development partnership
PECET: Program for the study and control of tropical diseases
R&D: Research and development
SMEs: Small and medium enterprises
TRIPS: Trade-related aspects of intellectual property
UMICs: Upper-middle-income countries
WHO: World Health Organization
WTO: World Trade Organization

## Footnotes

1 The article contains extracts from [2, 17, 39].

2 ICTRP definitions: Funder: "Source(s) of Monetary or Material Support: Major source(s) of monetary or material support for the trial (e.g. funding agency, foundation, company, institution)". "Primary sponsor: The individual, organization or company responsible for initiating, managing and/or financing a trial. The Primary Sponsor may or may not be the main funder". "Secondary sponsor(s): Additional individuals, organizations or other legal persons, if any, that have agreed with the primary sponsor to take on responsibilities of sponsorship" (WHO, ICTRP, WHO data set).

3 Information from the scoping interviews was not used for the list of the top LMICs, as there were few interviews conducted and several focused in a specific country or region.
